# Comparison of patient-led, fibromyalgia-orientated physical activity and a non-specific, standardised 6-month physical activity program on quality of life in individuals with fibromyalgia: a protocol for a randomised controlled trial

**DOI:** 10.1186/s13063-020-04730-3

**Published:** 2020-09-17

**Authors:** T. Rulleau, L. Planche, F. Etcheverrigaray, A. Dorion, N. Kacki, M. Miot, A. Liaigre, Y. Ganem, A. Schmidt, F. Taddéi, S. Acapo, J. Nizard, Y. M. Pluchon

**Affiliations:** 1 Unité de Recherche Clinique, CHD-Vendée, La Roche-sur-Yon, France; 2 Hopital Bel Air, Service Pharmacie, Courcoué sur Logne, France; 3 Groupe Associatif Siel Bleu, Strasbourg, France; 4 Centre d’Etude et de Traitement de la Douleur, CHD-Vendée, La Roche-sur-Yon, France; 5 Laboratoire Thérapeutique EA 3826, CHU Nantes et cabinet de kinésithérapie, 5 rue Nina Simone, 44000 Nantes, France; 6 Service Douleur Soins Palliatifs et de Support, Médecine intégrative, Unité de Recherche Clinique Douleur et Neurochirurgie, CHU Nantes, et UMR INSERM SPHERE, Nantes, France

**Keywords:** Activity, Fibromyalgia, Myofascial pain syndromes, Musculoskeletal diseases, Rheumatic diseases, Pain, Quality of life

## Abstract

**Background:**

Exercise has been shown to significantly improve pain and function in individuals with fibromyalgia. Research into the effectiveness of exercise is often based on standardised exercise programmes that are chosen by the investigating clinical research team. However, such programmes may not necessarily be appealing to the participating patients. Furthermore, in addition to being taught exercises, patients with chronic conditions like fibromyalgia also need to learn to manage their condition themselves and so be actively involved in their treatment. The primary aim of this study is to compare the effects of two, 6-month physical activity programs on quality of life in patients with fibromyalgia. One group followed a patient-led, fibromyalgia-orientated programme (experimental) whilst the control group followed a standard, general exercise programme.

**Methods:**

This protocol is an open-label, two-centre, randomised, controlled superiority trial. Two treatment arms will be compared: an experimental group (patient-led, fibromyalgia-orientated exercise) and a control group (general exercise program). The control group will participate in the exercise programme currently provided in our centre, which involves general, group exercise for patients with various pathologies. The experimental group will be taught the principles of exercise specifically for fibromyalgia during a one-to-one coaching session. They will then be guided in the choice of one or several types of exercise that they enjoy. They will be instructed to perform the exercise according to the recommendations for exercise in fibromyalgia with regard to intensity, duration and frequency. The protocol will last for 6 months; participants will then be followed-up for a further 6 months. They will also be encouraged to continue exercising after the end of the protocol.

Outcomes will be evaluated at baseline, 6 and 12 months. The primary outcome will be quality of life (Fibromyalgia Impact Questionnaire) and the secondary outcomes will include measures of pain (including a visual analogue scale and the neuropathic characteristics of the pain), depression (Hospital Anxiety and Depression Scale), kinesiophobia (Tampa scale of kinesiophobia) and adherence (Polar OH1 heart rate monitor).

**Discussion:**

The results of this study will show if patient-led, fibromyalgia-orientated exercise is more effective than a general exercise programme on fibromyalgia-related outcomes, including quality of life, and on adherence to continued exercise.

**Trial registration:**

ClinicalTrials.gov NCT03895086. Registration no. 2018-A02881-54. Registered on 29 March 2019

## Administrative information

**Table:** 

Title {1}	Comparison of patient-led, fibromyalgia-orientated physical activity and a non-specific, standardised 6-month physical activity program on quality of life in individuals with fibromyalgia: a protocol for a randomised controlled trial.
Trial registration {2a and 2b}	ClinicalTrials.gov Identifier: NCT03895086, registration n°: 2018-A02881-54
Protocol version {3}	Version 3 (02.10.2019)
Funding {4}	Internal funding from Centre Hospitalier Départemental Vendée
Author details {5a}	Thomas Rulleau, Lucie Planche, François Etcheverrigaray, Agnes Dorion, Nicolas Kacki : Unité de Recherche Clinique, CHD-Vendée, France Margot Miot, Aline Liaigre: Groupe Associatif Siel Bleu, Strasbourg, France Acapo Sessi, Laboratoire thérapeutique EA 3826, CHU Nantes et cabinet de kinésithérapie, 5 rue Nina Simone, 44000 Nantes, France Julien Nizard: Service Douleur Soins Palliatifs et de Support, Médecine intégrative, Unité de Recherche Clinique Douleur et Neurochirurgie, CHU Nantes, et UMR INSERM SPHERE, France Yves-Marie Pluchon: Centre d’Etude et de Traitement de la Douleur, CHD-Vendée, France
Name and contact information for the trial sponsor {5b}	Agnes Dorion, agnes.dorion@chd-vendee.fr CHD VENDEE – Bd Stéphane Moreau 85925 LA ROCHE-SUR-YON Cedex 9 France
Role of sponsor {5c}	chd-vendée is involved in study design; collection, management, analysis, and interpretation of data.

## Background and rationale {6a}

Fibromyalgia is a common, costly and controversial condition [1, 2], with a prevalence of 2 to 4% [1]. Two studies in France estimated the prevalence to be between 1.4 and 1.8% [3, 4]. The incidence is higher in women (11.3%) than in men (6.9%) [1]. Fibromyalgia is a complex syndrome that encompasses a large range of symptoms and functional limitations [1, 2, 5] and reduces quality of life [4, 6, 7]. Two factors have been shown to improve quality of life in individuals with fibromyalgia: diagnosis of the condition and initiation of treatment [8]. Treatment usually involves a multimodal approach, encompassing functional, psychological, pharmacological and social-professional [1] aspects of health. The most frequently used non-pharmaceutical treatments are as follows: pain education, cognitive behavioural therapy, composite therapy (education or counselling associated with physical exercise) and aerobic exercise [1].

Exercise has been shown to significantly improve pain and function in individuals with fibromyalgia [9–11]. To date, strength training and aerobic exercise carried out on land or as hydrotherapy have all been shown to be equally effective [9, 11]; therefore, current guidelines recommend both aerobic exercise and strength training [2, 10]. The recommended dose of aerobic exercise is 20 min (or 10 min twice), two to three times per week (at 70–80% of the maximal theoretical heart rate) and eight repetitions of each strengthening exercise two to three times per week [9, 10]. However, it has been suggested the dose of physical exercise must be adapted to the individual’s needs [12].

In research into the effectiveness of exercise, the type of exercise and dose is usually standardised. This means that the type of physical activity carried out is usually not determined by the patient but by the clinical research team, and it may therefore not actually be appealing to the individual. Furthermore, fibromyalgia is a chronic condition and, for chronic medical conditions [13], as well as being taught to exercise, patients must also be taught to manage the condition themselves and to be actively involved in their treatment. This is called life-style coaching. A cohort pilot study of 10 individuals with fibromyalgia [14] showed a 37% improvement in quality of life measured with the Revised Fibromyalgia Impact Questionnaire following life-style coaching sessions with health professionals.

In France, the current treatment for patients with fibromyalgia involves pharmacological treatment, physical therapy and psychological counselling. In both of the French hospitals that will participate in the current study, patients are given the opportunity to participate in an adapted physical activity program provided by an association called “Siel Bleu”. This association provides non-specific exercise classes for individuals with varying pathologies (stroke, diabetes, obesity etc.), with minimal equipment (due to material constraints). Although this exercise program provides many general benefits, it does not meet the current recommendations for physical activity in fibromyalgia [9].

As part of an effort to improve the management of patients with fibromyalgia, we have decided to continue the work of Hackshaw et al. [14] and use a pragmatic approach to exercise as part of the management of fibromyalgia. We wish to evaluate patient-led physical activity that is orientated towards the management of fibromyalgia. These activities will be supported by health professionals initially, although support will be gradually reduced to promote patient autonomy. In this way, we hope that patients will take leadership of their physical activity [14].

It is anticipated that this pragmatic solution will progressively increase patient participation in physical activity and improve exercise compliance, thus resulting in a greater medium- and long-term improvement in quality of life. The aim of this study, named FibrAPSpé (Fibromylagie et Activité Physique Spécifique), is, therefore, to develop a pragmatic approach that should lead to advances in knowledge and offer real-world treatment solutions.

### Objectives {7}

#### Hypothesis

We hypothesise that in comparison with the standard, general exercise program provided by Siel Bleu, a patient-led, fibromyalgia-orientated physical activity program will improve quality of life in individuals with fibromyalgia in both the medium- (6 months) and long-term (12 months).

#### Primary objective

The primary objective is to compare the effect of patient-led, fibromyalgia-orientated physical activity and a non-specific, standardised 6-month physical activity program on quality of life in patients with fibromyalgia.

#### Secondary objectives

The secondary objectives are to compare the effect of a patient-led, fibromyalgia-orientated physical activity and a non-specific, standardised 6-month physical activity program in patients with fibromyalgia on the following variables:

1. Change in pain intensity
2. The presence of, and change in, neuropathic pain characteristics
3. Isochrony between a physical movement and an imagined movement [15, 16]
4. Change in anxiety and depression
5. Change in pain on muscle compression
6. Change in symptom severity
7. Change in compliance with physical activity, and percentage achievement of objectives
8. Change in lean muscle mass
9. Change in kinesiophobia
10. Change in quality of life from baseline to 12 months
11. Continuation of physical activity after the program
12. Change in medication

### Trial design {8}

**Fig. 1:**
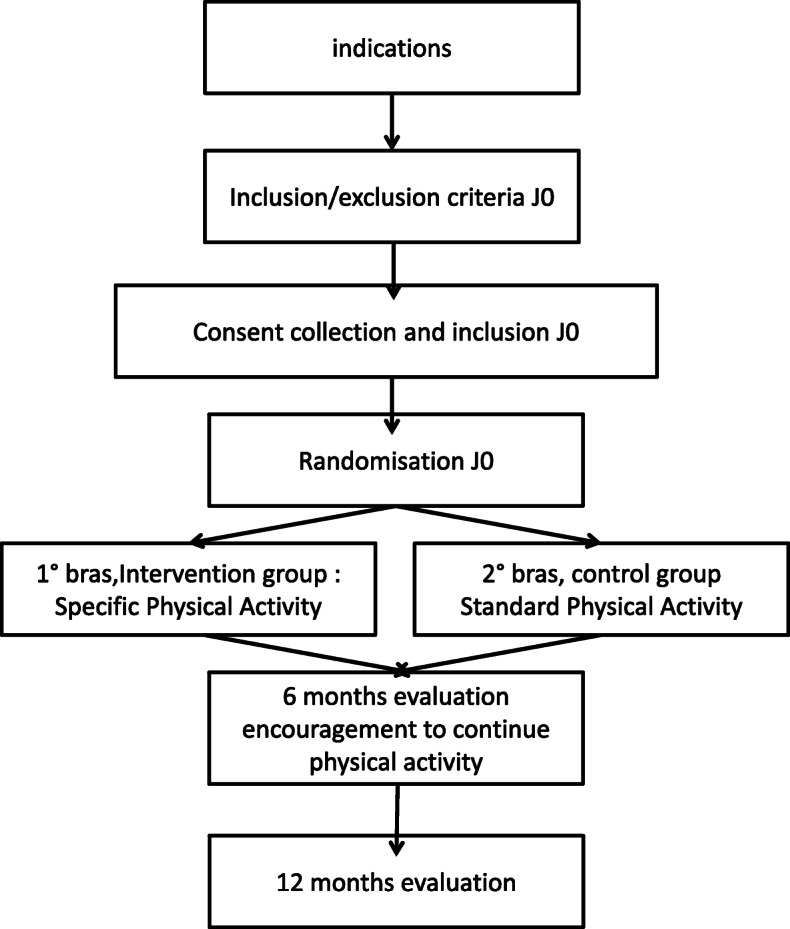
Flow diagram of the trial design

We will conduct an open-label, two-centre, randomised, controlled superiority trial with two parallel groups and a primary endpoint of change in quality of life from baseline (0 month) to 6 months (Fig. 1). Study participants will be randomised to receive either the control treatment (current care) or the experimental treatment (patient-led, fibromyalgia-orientated physical activity). Randomisation will be performed as block randomisation with a 1:1 allocation.

This article has been written in accordance with the SPIRIT (Standard Protocol Items: Recommendations for Interventional Trials) guidelines [17].

## Methods: participants, interventions and outcomes

### Study setting {9}

The study will be carried out in two centres: the Centre Hospitalier Départemental de la Vendée and the Centre Hospitalo-Universitaire de Nantes.

### Eligibility criteria {10}

#### Inclusion criteria

- Men or women over 18 years of age
- Fulfil the “2016 Revisions to the 2010/2011fibromyalgia diagnostic criteria” from the American Rheumatology Association [2]
- Score ≤ 59/100 on the revised Fibromyalgia Impact Questionnaire (FIQR) showing a mild or moderate impact of fibromyalgia on health [6]
- Able to be followed for 12 months
- Able to carry out the activities proposed by “Siel Bleu”, both physically and in terms of personal/professional schedule
- Own a smartphone and/or a computer (Mac or PC) with an internet connection allowing the use of a monitoring application related to the activity tracker
- Able to understand the protocol

#### Exclusion criteria

- Having participated in an interventional trial within the three months prior to inclusion
- Pregnant or breast feeding
- Under legal guardianship
- Unable to follow the protocol, based on the judgement of the investigator, or unwilling to use digital applications
- Contraindications to physical activity
- Having already participated in a physical activity program proposed by “Siel Bleu”

### Who will take informed consent? {26a}

In accordance with current French legislation, written informed consent will be obtained from each patient prior to their participation in the study. Consent will be acquired by the investigator during the inclusion visit that occurs beforehand.

If a patient refuses to give consent, the investigator will still treat them according to the current recommendations. The patient can decide to end their participation at any time, with no consequences on the quality of the subsequent care they will receive. To end their participation, patients will be told that they must inform their investigating doctor about their decision to withdraw. Once a patient has withdrawn consent, any personal data already collected may still be processed, unless they also provide written refusal for this.

### Additional consent provisions for collection and use of participant data and biological specimens {26b}

Not applicable; there is no need for additional consent for participant data. There is no biological collection.

## Interventions

### Explanation for the choice of comparators {6b}

The treatment provided within our centres in terms of physical activity does not meet the current recommendations for physical activity in fibromyalgia. We wished to determine if a pragmatic, patient-led approach, based on recommendations and that could realistically be implemented in our centres would be more effective.

### Intervention description {11a}

Flow diagram of the trial design is illustrated in Fig. 1.

#### Control group

The control group will follow the “Siel Bleu” non-specific, group exercise class once per week for 6 months, led by an adapted physical activity coach.

A typical session involves endurance training with a 10-min warm-up, 40 min of interval training and 10 min of cool-down, once per week. Participants can connect to the association’s website and follow a complementary exercise program if they wish. This complementary physical activity is optional and consists of exercises provided by an internet application and performed alone by the individual.

Participants in the control group will be asked to wear the activity-tracking device during all supervised or autonomous exercise sessions.

#### Experimental group

The experimental group will undergo 1 h of one-to-one coaching by an adapted physical activity coach followed by eight 30-min sessions of telephone coaching, alternating with a physiotherapist and an adapted physical activity coach over a period of 6 months. Physiotherapists and physical activity coaches will attend a training session in order to ensure standardise practices. The aim of the one-to-one coaching session is to determine the participant’s choice of physical activity(s).

The face-to-face coaching session will include:

- Health and physical activity education
- Explanation of the recommendations for aerobic exercise for fibromyalgia, which participants will be strongly encouraged to follow:
  - ◦ Duration: 20 min (or twice 10 min)
  - ◦ Frequency: two to three times per week
  - ◦ Intensity: at 70–80% of the maximal theoretical heart rate
  - ◦ The dose of physical exercise must be adapted to the individual’s needs
- Recording of the participant’s expectations using a standardised form, including personal and professional goals
- Discussion of the choice of physical activity(s) and the possibility of progressing or changing during the protocol
- Determination of realistic goals to be achieved over the 6 months, in line with the patient’s choice and the guidelines
- Determination of short and medium-term objectives
- Determination of maximal theoretical heart rate and explanation of subjective signs that 70–80% has been reached during exercise (e.g. patients should just be able to talk)

The telephone coaching sessions will include:

- Recording of achievements, problems and expectations
- Discussion regarding the management of physical activity, any difficulties, implementation of short and medium-term objectives
- Motivation to continue and progress exercise and to exercise at 70–80% of maximal theoretical heart rate

#### Exercise-tracking device: Polar OH1 bracelet

The Polar OH1 is an optical heart rate (HR) monitor bracelet worn on the upper arm (©Polar Electro). Waterproof and suitable for all sports, it has been previously demonstrated to provide accurate heart rate data during moderate- and high-intensity exercise [18]. The device connects to the manufacturer’s website via a smartphone or computer allowing HR information and wearing times to be stored. These data will be used to calculate the intensity, duration and frequency of each participant’s exercise each week.

### Criteria for discontinuing or modifying allocated interventions {11b}

The criteria for discontinuation are death or withdrawal of patient consent prior to publication of results. The patient can also object to the use of their data at any time up until submission for publication.

### Strategies to improve adherence to interventions {11c}

#### Activity monitoring and exercise surveillance after 6 months

At the end of 6 months, participants in both groups will be encouraged to continue with their physical activities. Compliance will be evaluated using the Polar OH1 activity tracker (©Polar Electro) to record physical activity frequency, duration and intensity for 12 months in total. Patients in both groups will be provided with a Polar OH1, shown how to use it and asked to wear it during all physical activity sessions. After each session, they will be asked to connect to the device manufacturer website via smartphone or computer to upload information. For experimental group participants, data will be analysed weekly to determine if they are exercising as recommended. If necessary, the participant will then be motivated to continue/increase during the next telephone coaching session.

Data collected from the control group will be used to compare the levels of physical activity at 6 months and 12 months between groups.

### Relevant concomitant care permitted or prohibited during the trial {11d}

Both groups will receive standard care, including medical consultations, nursing and psychological consultations, physical therapy and medication.

### Provisions for post-trial care {30}

At the end of the protocol but before the end of the study (i.e. between 6 and 12 months), participants in both groups will be able to choose to undergo physical activity coaching with either an out-patient physiotherapist or an adapted physical activity coach from “Siel Bleu”. Participants could also join in (or continue, for those in the control group) with the activity program run by “Siel Bleu” which is the current standard care in our centres. Patients will be encouraged to continue to wear their activity tracker during all physical activity sessions.

In addition, a specific insurance policy has been taken out to cover any damage resulting from participation in this study.

### Outcomes {12}

#### Primary outcome measure

The primary outcome measure is change in FIQR from baseline to 6 months.

#### Secondary outcome measures

The following outcomes will be compared between the two treatment arms:

1. Change in pain intensity measured on a visual analogue scale from baseline to 6 months, and baseline to 12 months [19]
2. The proportion of patients with neuropathic pain characteristics measured on the Douleur Neuropathique 4 scale (DN4) ≥ 4 at baseline, 6 months and 12 months [20]
3. Change in isochrony between a physical movement and an imagined movement [15, 16]. Although genetic, stress-related and environmental causes of fibromyalgia have been reported [1], sensitization of the central nervous system appears to be a key aspect of the condition [1]. Isochrony between physically executed and mentally simulated movement provides an indication of disruption between planning and motor execution in relation to central sensitization [16].
4. Change in Hospital Anxiety and Depression scale score from baseline to 6 months, and baseline to 12 months [21]
5. Change in widespread pain index score from baseline to 6 months, and baseline to 12 months [2, 22]
6. Change in symptom severity scale score from baseline to 6 months, and baseline to 12 months [2, 22]
7. Compliance with physical activity using activity trackers (Polar OH1).
8. Change in lean muscle mass (Tanita InnerScan 50) from baseline to 6 months, and baseline to 12 months
9. Change in Tampa Scale for Kinesiophobia from baseline to 6 months, and baseline to 12 months
10. Change in FIQR (quality of life) from baseline to 12 months
11. Continued physical activity between 6 and 12 months (measured using the activity tracker).
12. Change in Medication Quantification Scale Version III

### Participant timeline {13}

All assessments will be carried out at baseline (0 months), after 6 months (6 months: end of the protocol) and again at 12 months (12 months: end of the study) (Table 1).

**Table 1:** Study schedule of enrolment, interventions and assessments

Timepoint	Enrolment	0–6 months	6 months	6–12 months	12 months
Eligibility screening	x				
Written consent	x				
Patient history	x				
Randomisation	x				
Clinical examination and questionnaires^1^	x		x		X
**Experimental group**					
One-to-one coaching (1 session)		x			
Telephone coaching (8 sessions)		x			
Physical activity with activity trackers		x	x	x	X
Encouragement to continue physical activity					
**Control group**					
Usual treatment (group exercise class)		x			
Analgesic therapy	x		x		X
Adverse events		x	x	x	X

^1^: VAS, DN4, HAD, WPI, SSS, impedancemetry, Tampa, FIQR, EQ-5D-5L

During the trial inclusion visit, eligible participants will be provided with an activity-tracking device (see the “Exercise-tracking device: Polar OH1 bracelet” section) and shown how to use it.

### Sample size {14}

The study by Hackshaw et al. [14] used inclusion criteria that were similar to those of the present study; the mean FIQR at baseline for the 10 patients included was 49.4 ± 13.8 (mean + SD). We consider that a 7-point difference between the two treatment arms at 6 months would be clinically significant. The sample size required to show a between-group difference of 7 points in the FIQR with a power of 0.80 and an alpha risk of 0.05 is 114 patients. In order to ensure this power is achieved, 10% more patients will be included, thus a total of 126 patients.

### Recruitment {15}

A study of patient files in the Vendée Hospital Pain Clinic and the Siel Bleu Association found that an average of 4 new patients with fibromyalgia are registered per week; an equivalent of around 200 new patients each year.

The Nantes University Hospital has estimated that, based on their patient population and the resources necessary for the project, they could recruit 24 eligible patients per year. Assuming an attrition or refusal rate of 20%, this would represent a combined total of 179 patients a year from both hospital sites. In order to ensure the target sample size (*n* = 126) is reached, the study period will last 18 months.

## Assignment of interventions: allocation

### Sequence generation {16a}

Participants will be randomised in a 1:1 ratio to either the control or the experimental group by a computer-generated randomisation schedule using permuted blocks of random sizes.

### Concealment mechanism {16b}

Participants will be randomly allocated into either group using Ennov Clinical, an online, central randomisation service (Ennov Clinical [23]). Connection will require a login, password and study number which will be provided by the data manager from the Vendée Hospital Research Department. The following patient information will be uploaded to Ennov Clinical for the randomisation:

- First name and surname initials
- Month and year of birth
- Fulfilment of the inclusion criteria (yes)

### Implementation {16c}

After confirmation that the patient is eligible for inclusion in the study, their information will be uploaded to Ennov Clinical by the investigator for randomisation. An inclusion number will be automatically attributed with the randomisation. Email confirmation of inclusion will be sent to the person carrying out the randomisation as well as all others concerned (principal investigator and his local team). The randomisation list will be generated by the biostatistician-methodologist of the Vendée Hospital Research Department.

## Assignment of interventions: blinding

### Who will be blinded {17a}

Neither the patients nor the coaches or the medical team will be blinded to group allocation.

### Procedure for unblinding if needed {17b}

Not applicable, this study is open

## Data collection and management

### Plans for assessment and collection of outcomes {18a}

The FIQR is a revised version of the FIQ [24]. The FIQR has good psychometric properties and there is a good correlation with the original FIQ [24] which will allow comparison of the results with other studies that have used the older version.

Professionals trained in clinical evaluation and completion of the questionnaires will carry out the evaluations. Most of the proposed tests are performed in routine practice. The exceptions are the Tampa self-test and the isochrony assessment. In order to standardise these evaluations, training will be provided by a research practitioner who is an expert in this field (T.R.).

### Plans to promote participant retention and complete follow-up {18b}

Patients will be followed up during their usual algology consultations.

### Data management {19}

All data will be anonymised: patients will receive an identification number based on their initials and the order and centre of their inclusion. The number will be generated by Clinsight software, an online, electronic case report management system (eCRF; Ennov Clinical). This code will be the only patient identification information used in the eCRF; all documents used in the eCRF will also be coded in this manner.

One eCRF will be created for each patient and will contain all information required for analysis of the exercise protocol data. The eCRF will indicate if the protocol has been respected, if there are any discrepancies and all the data required for the statistical analyses.

The investigators or research assistants responsible for completing the eCRF at each centre will be noted in the investigator’s folder. The investigator is responsible for the accuracy and quality of all the data stored in the eCRF. Data will be verified as they are entered into the eCRF and any modifications to entries will be validated by the investigator; this change will be recorded and justification may also be entered as a comment. Data will be stored at the participating sites for 15 years.

An eCRF patient code correspondence table will be made for all patients at both participating centres. It will contain the both the patient’s name and eCRF code and will be used in cases of erroneous or missing data. The correspondence table will be held by the principal investigator at each centre in a secure place and no clinical data will be recorded on it.

### Confidentiality {27}

The study will be conducted in accordance with the recommendations of the French Data Protection Authority and data protection regulations [25]. All source data will be kept in the investigating centre. The electronic database will be anonymous and will be kept for 15 years according to Good Clinical Practice [25].

### Plans for collection, laboratory evaluation and storage of biological specimens for genetic or molecular analysis in this trial/future use {33}

Not applicable as no biological specimens will be collected as part of this trial

## Statistical methods

### Statistical methods for primary and secondary outcomes {20a}

#### Primary outcome

Baseline characteristics will be described for each arm.

Summary statistics provided for continuous variables will include the number of subjects (*n*), means, medians, standard deviations or standard errors, minima and maxima. For categorical variables, the frequencies and percentages will be provided.

Quality of life will be rated using the FIQR at baseline, 6 months and 12 months. Changes in scores from baseline to 6 months, and from baseline to 12 months will be plotted graphically.

The primary outcome will be analysed using a linear mixed effect model that will account for: score at baseline, the time effect and time*treatment interaction as fixed effects and a random patient effect.

#### Secondary outcomes

All secondary outcomes will be analysed using the same method as for the primary outcome. Linear mixed effects models will account for baseline scores, the time effect, time*treatment interactions and a random patient effect.

The presence of neuropathic pain (DN4 ≥ 4), lean muscle mass and kinesiophobia will be analysed using a generalised linear model accounting for baseline values, the time effect, time*treatment interaction and a random patient effect.

The number of hours and intensity of physical activity will be collected monthly for 12 months. Each month, the percentage success of the objectives will also be calculated. We set the goal at 60 min of physical activity per week carried out at a minimum of 70% of the theoretical maximum heart rate. The patient’s physical activity time beyond this frequency will allow us to calculate the percentage of time the goal is achieved. The mean percentage of success will be plotted on a graph and treatment arms will be compared using a mixed linear regression model that accounts for repeated measured.

The main analysis will be carried out in intention to treat and thus will include all patients who are randomised.

A sensitivity analysis will be carried out per protocol and will include the patients who were randomised and for whom no major deviation from the protocol occurred.

A meeting will be organised to determine the severity of any deviations from the protocol that occur.

#### Exploratory criterion

Change in the FIQR score will be evaluated using a mixed linear model accounting for the group and the repeated data as well as isochrony at baseline to determine the capacity of isochrony to predict change in FIQR.

### Interim analyses {21b}

None planned

### Methods for additional analyses (e.g. subgroup analyses) {20b}

None planned

### Methods in analysis to handle protocol non-adherence and any statistical methods to handle missing data {20c}

Any missing data and the reason for the missing data will be described in each group. For the analysis of the FIQ, a multiple imputation model will be applied. For the other criteria, no imputation will be performed.

### Plans to give access to the full protocol, participant level-data and statistical code {31c}

The protocol is available on request from the CHD-Vendée. At this time, there are no plans to provide access to the participant level-data and statistical code.

## Oversight and monitoring

### Composition of the coordinating centre and trial steering committee {5d}

The coordination centre is a clinical research unit that provides supports to the study coordinator. It is composed of a project manager, a data analyst and a data monitoring team.

### Composition of the data monitoring committee, its role and reporting structure {21a}

Data monitoring will be carried out by an employee of the sponsor department of the clinical research unit of Vendee Hospital to ensure that the eCRP are completed accurately. Data quality control will be regularly undertaken at each investigating site to ensure that the eCRP are accurately completed. The protocol is considered to be Risk Level A (‘low or negligible risk’); it will be monitored to ensure that:

- Informed consent has been received from each participant
- The clinical centre has complied with the requirements of the protocol
- The data are complete and accurate
- All original patient information and documentation, such as medical notes, appointment cards or laboratory result sheets, are present and complete.

### Adverse event reporting and harms {22}

The trial may be temporarily interrupted for any participant at the discretion of the lead investigator if a serious adverse event caused by the study protocol is suspected. A procedure for such events has been described in accordance with current French regulations.

### Frequency and plans for auditing trial conduct {23}

An inspection or audit may be carried out by French National Agency for the Safety of Medicines and Health Products. In this case, the sponsor and/or the participating centres will allow the inspectors and/or auditors full access to the data.

The medical data for each patient will be transmitted to the sponsor or to any person habilitated by the sponsor, or the health authorities, and confidentiality will be fully respected.

The sponsor and guardianship authorities may request direct access to any patient’s file in order to check that procedures are being followed and/or that data are being collected as defined in the protocol, within the limits authorised by laws and regulations [25].

### Plans for communicating important protocol amendments to relevant parties (e.g. trial participants, ethical committees) {25}

Substantial changes will be added on ClinicalTrials.gov and other online registrations (WHO register).

### Dissemination plans {31a}

The study coordinator will communicate the results of the study to the investigators and to any participants who stated that they wished to be kept informed. The results will only be disseminated once the study is finished. This will be either the last evaluation visit of the final participant or, if applicable, following early termination of the study. Results from this work will be communicated to health professionals and expert groups via publications, oral communications or posters in medical congresses.

## Discussion

The primary aim of this study, named FibrAPSpé (Fibromyalgie et Activité Physique Spécifique), was to compare the effects of two, 6-month physical activity programs on quality of life in patients with fibromyalgia and other fibromyalgia-related variables. This study will provide important information regarding the effects of patient-led physical activity on continued adherence to an exercise programme, which is essential if physical activity is to be effective [8, 10, 11, 14].

The medium-term results (6 months) will show if the patient-led, fibromyalgia-orientated physical activity is more effective than the general exercise program in improving fibromyalgia related outcomes such as pain, depression, kinesiophobia and quality of life.

The long-term results (12 months) will show if continued adherence to exercise is greater in the group of participants who engaged in their own choice of physical activity compared with those who followed an imposed and general exercise program. The results will also indicate if continued adherence impacts on quality of life.

The results of this study will therefore provide a guide for clinicians involved in the management of patients with fibromyalgia regarding the most appropriate methods for patients to engage in physical activity.

The main limitation of this study is that it cannot be double blind. This classic limitation in rehabilitation is the direct result of human intervention related to the study.

## Trial status

Version of protocol number 2 filed March 29, 2019

Recruitment began on February 2020 and is expected to be completed in September 2021 to include 126 patients.

## Supplementary information

**Additional file 1.**

**Additional file 2.**

## Data Availability

No data have yet been acquired so data sharing is not applicable to this article. The possibility of making the raw data available has been planned. If these data are used for subsequent publications, the coordinator must be named as a co-author and his affiliation must be specified.

## Abbreviations

DN4: Douleur Neuropathique 4 questionnaire
eCRF: Electronic case report form
FibrAPSpé: Fibromylagie et Activité Physique Spécifique
FIQR: Fibromyalgia Impact Questionnaire Revised
SPIRIT: Standard Protocol Items: Recommendations for Interventional Trials
SSS: Severity Symptom Scale
WPI: Widespread pain index
