# Supplementary material for: Comparison of patient-led, fibromyalgia-orientated physical activity and a non-specific, standardised 6-month physical activity program on quality of life in individuals with fibromyalgia: a protocol for a randomised controlled trial
Source: Trials. 2020 Sep 17;21:800. doi: 10.1186/s13063-020-04730-3 (PMC7499844; doi:10.1186/s13063-020-04730-3)
Supplement: Supplementary file 1 — Additional file 1. [file 13063_2020_4730_MOESM1_ESM.pdf]

ANNEXE 3: DONNEES RECUEILLIES

Inclusion :
- Initiales
- Sexe
- Age
- Statut marital
- Antécédents
- Date de diagnostic de la maladie
- Activité professionnelle
- Arrêt ou non de travail
- Activité physique : pratique actuelle (temps) + type d'activité
- Questionnaire FIQR
- EVA
- Questionnaire DN4
- Durée des mouvements exécutés physiquement et simulés mentalement.
- Hospital Anxiety and Depression scale (HAD)
- Widespread pain Index (WPI)
- Echelle de sévérité des symptômes (SSS)
- % et poids de masse maigre mesurée sur la balance d'impédancemétrie
- Questionnaire de Tampa
- EQ-5D-5L

1 mois/ 2 mois/ 3 mois/ 4 mois/ 5 mois :
- Durée en minutes par mois entre 50 et 60, entre 60 et 70, entre 70 et 80, entre 80 et 90, et entre 90et 100% de la fréquence cardiaque maximale théorique.

6 mois :
- Questionnaire FIQR
- EVA
- Questionnaire DN4
- Durée des mouvements exécutés physiquement et simulés mentalement.
- Hospital Anxiety and Depression scale (HAD)
- Widespread pain Index (WPI)
- Echelle de sévérité des symptômes (SSS)
- Durée en minutes par mois entre 50 et 60, entre 60 et 70, entre 70 et 80, entre 80 et 90, et entre 90et 100% de la fréquence cardiaque maximale théorique.
- % et poids de masse maigre mesurée sur la balance d'impédancemétrie
- Questionnaire de Tampa
- EQ-5D-5L
- Consultation : nombre de consultations en lien avec la fibromyalgie ou ses conséquences chez le médecin généraliste, et/ou médecin spécialiste,
- Hospitalisation en lien avec la fibromyalgie ou ses conséquences (durée et service)
- Activité professionnelle (arrêt de travail ou non + date de reprise si applicable)

7 mois/ 8 mois/ 9 mois/ 10 mois/ 11 mois :
- Durée en minutes par mois entre 50 et 60, entre 60 et 70, entre 70 et 80, entre 80 et 90, et entre 90et 100% de la fréquence cardiaque maximale théorique.

12 mois :
- Questionnaire FIQR
- EVA
- Questionnaire DN4

- Durée des mouvements exécutés physiquement et simulés mentalement.
- Hospital Anxiety and Depression scale (HAD)
- Widespread pain Index (WPI)
- Echelle de sévérité des symptômes (SSS)
- Durée en minutes par mois entre 50 et 60, entre 60 et 70, entre 70 et 80, entre 80 et 90, et entre 90et 100% de la fréquence cardiaque maximale théorique.
- % et poids de masse maigre mesurée sur la balance d'impédancemétrie
- Questionnaire de Tampa
- EQ-5D-5L
- Consultation : nombre de consultations en lien avec la fibromyalgie ou ses conséquences chez le médecin généraliste, et/ou médecin spécialiste,
- Hospitalisation en lien avec la fibromyalgie ou ses conséquences (durée et service)
- Continuité de l'activité physique
- Activité professionnelle (AT ou non + date de reprise si applicable)

Tout au long de l'étude :

- Troubles fonctionnels empêchant la réalisation d'une activité physique
- Dysfonctionnement du système de tracking d'activité
