# Supplementary material for: Comparison of patient-led, fibromyalgia-orientated physical activity and a non-specific, standardised 6-month physical activity program on quality of life in individuals with fibromyalgia: a protocol for a randomised controlled trial
Source: Trials. 2020 Sep 17;21:800. doi: 10.1186/s13063-020-04730-3 (PMC7499844; doi:10.1186/s13063-020-04730-3)
Supplement: Supplementary file 2 — Additional file 2. [file 13063_2020_4730_MOESM2_ESM.pdf]

CREATION DU PATIENT

Initiales **PATIENT** **Nom :** 1ère lettre **[__]** **Prénom :** 1ère lettre **[__]**

PAGE DE GARDE

« FibrAPSpé »

N° Centre : **[__][__]** **N° Patient** : **[__][__][__]**

PATIENT **Nom :** 1ère lettre **[__]** **Prénom :** 1ère lettre **[__]**

Date de recueil du consentement écrit: Date :

CRITERES D'INCLUSION

Tous les critères ci-dessous sont respectés : Oui/ Non

⚠ **Si la réponse est NON le patient n'est pas incluable.**

Si NON, merci de préciser :

1	Patient(e) majeur	Oui/ Non
2	Diagnostic de fibromyalgie (Häuser et al., 2017) :<br>4< WPI < 6 et SSS ≥9<br>**OU**<br>WPI ≥ 6 et SSS ≥5<br>**ET**<br>une douleur présente **au minimum** dans 4 des 5 régions du corps (5 régions = 4 cadrans + l'axe).	Oui/ Non
3	Patient(e) dont la FIQ-R est ≤59/100	Oui/ Non
4	Patient(e) pouvant être suivi durant 12 mois au sein du CETD	Oui/ Non
5	Patient(e) pouvant suivre les activités physiques proposées par « Siel Bleu », sur le plan physique et organisationnel	Oui/ Non
6	Patient(e) possédant un smartphone et/ou un ordinateur (mac/pc) avec une connexion internet permettant l'utilisation d'une application de suivi en lien avec le traqueur d'activité	Oui/ Non
7	Patient(e) ayant les capacités de comprendre le protocole et ayant donné son consentement pour participer à l'étude	Oui/ Non
8	Patient(e) bénéficiant d'une couverture sociale	Oui/ Non

CRITERES DE NON-INCLUSION

Y'a-t-il présence d'un critère de non-inclusion : Oui/ Non

⚠ **Si la réponse est OUI le patient n'est pas incluable.**

Si OUI, merci de préciser :

1	Patient(e) participant à un essai interventionnel dans les 3 mois précédents l'inclusion	Oui/ Non
2	Femme enceinte ou allaitante, ou ayant la possibilité de procréer sans moyen de contraception efficace	Oui/ Non
3	Patient(e) mineur, sous tutelle, curatelle ou privé de liberté	Oui/ Non
4	Patient(e) incapable de suivre le protocole, selon le jugement de l'investigateur, ou refusant l'utilisation des applications numériques,	Oui/ Non
5	Patient avec contre-indications à l'activité physique	Oui/ Non
6	Patient(e) ayant déjà suivi les activités physiques proposées par « Siel Bleu »	Oui/ Non

Visite d'inclusion (J0)

Date: I__I__I/I__I__I / I__I__I__I__I

RANDOMISATION

Cliquer sur « ACTION » situé en haut à gauche de l'écran puis cliquer sur « RANDOMISER »
Date de la randomisation : I__I__I/I__I__I/I__I__I__I__I

Bras de randomisation:

- Bras « Prise en charge classique non spécifique en groupe commun « multipathologique » »
- Bras « Prise en charge spécifique en coaching téléphonique « Patients fibromyalgiques » »

Pour toute randomisation non réalisée, merci de bien vouloir renseigner la raison dans la partie "Fin d'étude – Déviations au protocole".

CARACTERISTIQUES DEMOGRAPHIQUES

Sexe : Homme/ Femme

Date de naissance *(mois/ année)*: I__I__I/I__I__I__I__I

Statut marital: célibataire/ marié(e)/ veuf(ve)/ND

Antécédents traumatiques *(acte violent qui a provoqué une série de lésions et de troubles)* : Oui/ Non/ND
Si oui, préciser : mort soudaine et inattendue d'un proche/ témoin d'un décès/ témoin d'une agression/ / victime d'une agression/ victime d'un accident de la route/ victime d'un accident du travail/ autres
Si autres, préciser : …………………………………………

Date de diagnostic de la maladie : I__I__I/I__I__I/I__I__I__I__I

Activité professionnelle en cours: Oui/ Non/ND
Si oui, précisez : Agriculteurs exploitants/ Artisans, commerçants et chefs d'entreprise/ Cadres et professions intellectuelles supérieures/ Professions Intermédiaires/ Employés/ Ouvriers

Retraité : Oui/ Non/ ND

Arrêt de travail en cours: Oui/ Non/ ND

Si oui, date d'arrêt : I__I__I/I__I__I/I__I__I__I__I

CARACTERISTIQUES DEMOGRAPHIQUES (suite)

Activité physique en cours: Oui/ Non/ ND
Si oui, précisez :

Marche	Durée (en heures)/ semaine	Nb séances :	/semaine
Marche nordique	Durée (en heures)/ semaine	Nb séances :	/semaine
Running	Durée (en heures)/ semaine	Nb séances :	/semaine
Natation	Durée (en heures)/ semaine	Nb séances :	/semaine
Aquagym	Durée (en heures)/ semaine	Nb séances :	/semaine
Aquabike	Durée (en heures)/ semaine	Nb séances :	/semaine
Squash	Durée (en heures)/ semaine	Nb séances :	/semaine
Golf	Durée (en heures)/ semaine	Nb séances :	/semaine
Musculation	Durée (en heures)/ semaine	Nb séances :	/semaine
Vélo	Durée (en heures)/ semaine	Nb séances :	/semaine
Vélo elliptique	Durée (en heures)/ semaine	Nb séances :	/semaine
Rameur	Durée (en heures)/ semaine	Nb séances :	/semaine
Gymnastique holistique (yoga, taïchi, pilates, stretching, taiso…)	Durée (en heures)/ semaine	Nb séances :	/semaine
Art martial	Durée (en heures)/ semaine	Nb séances :	/semaine
Autres, précisez :	Durée (en heures)/ semaine	Nb séances :	/semaine
Autres, précisez :	Durée (en heures)/ semaine	Nb séances :	/semaine
Autres, précisez :	Durée (en heures)/ semaine	Nb séances :	/semaine

EVALUATION DE LA DOULEUR

EVA : I__I__I__I/100

MOUVEMENT EXECUTE/ IMAGINE

Durée du premier mouvement exécuté : I__I__I__I,I__I__I secondes
Durée du second mouvement exécuté : I__I__I__I,I__I__I secondes

Durée du premier mouvement simulé mentalement : I__I__I__I,I__I__I secondes
Durée du second mouvement simulé mentalement : I__I__I__I,I__I__I secondes

**QUESTIONNAIRE FIQ-R :** Réalisé/ Non Réalisé
1 – Etes-vous capable de :<br><br>Faire les courses :<br>- Toujours<br>- La plupart du temps<br>- De temps en temps<br>- Jamais<br>- ND<br><br>Faire la lessive en machine :<br>- Toujours<br>- La plupart du temps<br>- De temps en temps<br>- Jamais<br>- ND<br><br>Faire à manger :<br>- Toujours<br>- La plupart du temps<br>- De temps en temps<br>- Jamais<br>- ND<br><br>Faire la vaisselle à la main :<br>- Toujours<br>- La plupart du temps<br>- De temps en temps<br>- Jamais<br>- ND<br><br>Passer l'aspirateur :<br>- Toujours (<br>- La plupart du temps<br>- De temps en temps<br>- Jamais<br>- ND<br><br>Faire les lits :<br>- Toujours<br>- La plupart du temps<br>- De temps en temps<br>- Jamais<br>- ND<br><br>Marcher plusieurs centaines de mètres :<br>- Toujours<br>- La plupart du temps<br>- De temps en temps<br>- Jamais

- ND

Aller voir des amis ou de la famille :
- Toujours
- La plupart du temps
- De temps en temps
- Jamais
- ND

Faire du jardinage :
- Toujours
- La plupart du temps
- De temps en temps
- Jamais
- ND

Conduire une voiture :
- Toujours)
- La plupart du temps
- De temps en temps
- Jamais)
- ND

Au cours des 7 derniers jours :

2 – Combien de jour vous êtes-vous senti(e) bien ? I__I

3 – Combien de journées de travail avez-vous manqué à cause de votre fibromyalgie ? I__I

4 – Les jours où vous avez travaillé, les douleurs ou d'autres problèmes liés à votre fibromyalgie vous ont-ils gêné dans votre travail ? I__I__I,I__I

5 – Avez-vous des douleurs ? I__I__I,I__I

6 – Avez-vous été fatigué(e) ? I__I__I,I__I

7 – Comment vous êtes-vous senti(e) le matin au réveil ? I__I__I,I__I

8 – Vous êtes-vous senti(e) raide ? I__I__I,I__I

9 – Vous êtes-vous senti(e) tendu(e) ou inquiet(e) ? I__I__I,I__I (

10 – Vous êtes-vous senti(e) déprimé(e) ? I__I__I,I__I

Score global: ……../100

Si score > 59, INCLUSION A TORT

QUESTIONNAIRE DN4 : Réalisé/ Non Réalisé

1 – La douleur présente-t-elle une ou plusieurs des caractéristiques suivantes ?
- Brulure : Oui/ Non/ ND
- Sensation de froid douloureux: Oui/ Non/ ND
- Décharges électriques : Oui/ Non/ ND

2 – La douleur est-elle associée dans la même région à un ou plusieurs des symptômes suivants ?
- Fourmillements : Oui/ Non/ ND
- Picotements: Oui/ Non/ ND
- Engourdissements : Oui/ Non/ ND
- Démangeaisons : Oui/ Non/ ND

3 – La douleur est-elle localisée dans un territoire où l'examen met en évidence :
- Hypoesthésie au tact ? Oui/ Non/ ND
- Hypoesthésie à la piqûre ? Oui/ Non/ ND

4 – La douleur est-elle provoquée ou augmentée par :
- Le frottement ? Oui/ Non/ ND

Score du patient : …../10

QUESTIONNAIRE HAD : Réalisé/ Non Réalisé

1 – Je me sens tendu(e) ou énervé(e) :
- La plupart du temps
- Souvent
- De temps en temps
- Jamais
- ND

2 – Je prends plaisir aux mêmes choses qu'autrefois :
- Oui, tout autant
- Pas autant
- Un peu seulement
- Presque plus
- ND

3 – J'ai une sensation de peur comme si quelque chose d'horrible allait m'arriver :
- Oui, très nettement
- Oui, mais ce n'est pas trop grave
- Un peu, mais cela ne m'inquiète pas
- Pas du tout
- ND

4 – Je ris facilement et vois le bon côté des choses :
- Autant que par le passé
- Plus autant qu'avant

- Vraiment moins qu'avant
- Plus du tout
- ND

5 – Je me fais du souci :
- Très souvent
- Assez souvent
- Occasionnellement
- Très occasionnellement
- ND

6 – Je suis de bonne humeur :
- Jamais
- Rarement
- Assez souvent
- La plupart du temps
- ND

7 – Je peux rester tranquillement assis(se) à ne rien faire et me sentir décontracté(e) :
- Oui, quoi qu'il arrive
- Oui, en général
- Rarement
- Jamais
- ND

8 – J'ai l'impression de fonctionner au ralenti :
- Presque toujours
- Très souvent
- Parfois
- Jamais
- ND

9 – J'éprouve des sensations de peur et j'ai l'estomac noué :
- Jamais
- Parfois
- Assez souvent
- Très souvent
- ND

10 – Je ne m'intéresse plus à mon apparence :
- Plus du tout
- Je n'y accorde pas autant d'attention que je devrais
- Il se peut que je n'y fasse plus autant attention
- J'y prête autant d'attention que par le passé
- ND

11 – J'ai la bougeotte et n'arrive pas à tenir en place:
- Oui, c'est tout à fait le cas
- Un peu
- Pas tellement
- Pas du tout
- ND

12 – Je me réjouis d'avance à l'idée de faire certaines choses :
- Autant qu'avant
- Un peu moins qu'avant
- Bien moins qu'avant
- Presque jamais
- ND

13 – J'éprouve des sensations soudaines de panique:
- Vraiment très souvent
- Assez souvent
- Pas très souvent
- Jamais
- ND

14 – Je peux prendre plaisir à un bon livre ou à une bonne émission de radio ou de télévision :
- Souvent
- Parfois
- Rarement
- Très rarement
- ND

Score en lien avec l'anxiété : …../ 21
Score en lien avec la dimension dépressive : ….. / 21

QUESTIONNAIRE WPI : Réalisé/ Non Réalisé

Nombre de zones pour lesquelles le patient a ressenti de la douleur la semaine précédente : I__I__I/19

ECHELLE DE SEVERITE DES SYMPTOMES

Intensité des symptômes (au cours de la semaine passée): Réalisé/ Non Réalisé

1- Fatigue
- Pas de problème
- Problèmes légers ou modérés, généralement modérés ou intermittents
- Problèmes modérés, considérables, souvent présents et/ou à niveau modéré
- Problèmes importants, persistants, continus, handicapants au quotidien
- ND

2- Somnolence
- Pas de problème
- Problèmes légers ou modérés, généralement modérés ou intermittents
- Problèmes modérés, considérables, souvent présents et/ou à niveau modéré
- Problèmes importants, persistants, continus, handicapants au quotidien
- ND

3- Symptômes cognitifs

- Pas de problème
- Problèmes légers ou modérés, généralement modérés ou intermittents
- Problèmes modérés, considérables, souvent présents et/ou à niveau modéré
- Problèmes importants, persistants, continus, handicapants au quotidien
- ND

Score : ………/9

Nombre de symptômes associés (au cours de la semaine passée) : Réalisé/ Non Réalisé

☐ Douleur musculaire	☐Syndrome intestin irritable	☐Fatigue
☐Problème mémoire, concentration	☐Céphalées	☐Douleur/ crampes abdominales
☐Faiblesse musculaire	☐Engourdissement/ picotement	☐Vertiges
☐Insomnie	☐Dépression	☐Nervosité
☐Constipation	☐Douleur abdominale haute	☐Nausée
☐Douleur thoracique	☐Diarrhée	☐Bouche sèche
☐Vision floue	☐Fièvre	☐Démangeaisons
☐Sifflement respiratoire	☐Syndrome de Raynaud	☐Urticaire
☐Acouphènes	☐Vomissements	☐Brûlure thoracique
☐Aphtes buccaux	☐Perte ou modification du goût	☐Crise d'épilepsie
☐Yeux secs	☐Oppression respiratoire	☐Perte d'appétit
☐Eruption cutanée	☐Hypersensibilité au soleil	☐Troubles de l'audition
☐Ecchymoses faciles	☐Perte de cheveux	☐Mictions fréquentes
☐Douleurs à la miction	☐Spasmes de la vessie	

Score : ……………/3

Score total : ……………/12

QUESTIONNAIRE DE TAMPA : Réalisé/ Non Réalisé

1- J'ai peur de me blesser si je fais de l'activité physique :
- Fortement en désaccord
- Quelque peu en désaccord
- Quelque peu en accord
- Fortement en accord
- ND

2- Ma douleur ne ferait qu'intensifier si j'essayais de la vaincre :
- Fortement en désaccord
- Quelque peu en désaccord
- Quelque peu en accord
- Fortement en accord
- ND

3- Mon corps me dit que quelque chose ne va vraiment pas :
- Fortement en désaccord
- Quelque peu en désaccord
- Quelque peu en accord
- Fortement en accord

- ND

4- Si je faisais de l'activité physique, ma douleur serait probablement soulagée:
- Fortement en désaccord
- Quelque peu en désaccord
- Quelque peu en accord
- Fortement en accord
- ND

5- Les gens ne prennent pas mon état de santé au sérieux :
- Fortement en désaccord
- Quelque peu en désaccord
- Quelque peu en accord
- Fortement en accord
- ND

6- Mon accident a mis mon corps en danger pour le reste de mes jours :
- Fortement en désaccord
- Quelque peu en désaccord
- Quelque peu en accord
- Fortement en accord
- ND

7- La douleur signifie toujours que je me suis blessé(e) :
- Fortement en désaccord
- Quelque peu en désaccord
- Quelque peu en accord
- Fortement en accord
- ND

8- Même si quelque chose aggrave ma douleur, cela ne veut pas dire que c'est dangereux :
- Fortement en désaccord
- Quelque peu en désaccord
- Quelque peu en accord
- Fortement en accord
- ND

9- J'ai peur de me blesser accidentellement :
- Fortement en désaccord
- Quelque peu en désaccord
- Quelque peu en accord
- Fortement en accord
- ND

10- La meilleure façon d'empêcher que ma douleur s'aggrave est de m'assurer de ne pas faire des mouvements inutiles :
- Fortement en désaccord
- Quelque peu en désaccord
- Quelque peu en accord
- Fortement en accord
- ND

11- Je n'aurais pas tant que douleurs s'il ne se passait pas quelque chose de grave dans mon corps :

- Fortement en désaccord
- Quelque peu en désaccord
- Quelque peu en accord
- Fortement en accord
- ND

12- Bien que ma condition soit pénible, je serais mieux si j'étais physiquement actif(ve) :
- Fortement en désaccord
- Quelque peu en désaccord
- Quelque peu en accord
- Fortement en accord
- ND

13- La douleur m'indique quand arrêter de faires des activités physiques pour que je ne me blesse pas :
- Fortement en désaccord
- Quelque peu en désaccord
- Quelque peu en accord
- Fortement en accord
- ND

14- Il n'est pas prudent qu'une personne avec un état de santé comme le mien soit physiquement active :
- Fortement en désaccord
- Quelque peu en désaccord
- Quelque peu en accord
- Fortement en accord
- ND

15- Je ne peux pas faire tout ce qu'une personne normale peut faire parce que j'ai plus de risque de me blesser :
- Fortement en désaccord
- Quelque peu en désaccord
- Quelque peu en accord
- Fortement en accord
- ND

16- Bien qu'il y ait quelque chose qui me cause beaucoup de douleurs, je ne pense pas que ce soit vraiment grave :
- Fortement en désaccord
- Quelque peu en désaccord
- Quelque peu en accord
- Fortement en accord
- ND

17- Personne ne devrait être obligé de faire des exercices lorsqu'il (elle) ressent de la douleur :
- Fortement en désaccord
- Quelque peu en désaccord
- Quelque peu en accord
- Fortement en accord
- ND

Score : ……/68

EQ-5D-5L : Réalisé/ Non Réalisé

1- Mobilité
- Je n'ai aucun problème pour me déplacer à pied
- J'ai des problèmes légers pour me déplacer à pied
- J'ai des problèmes modérés pour me déplacer à pied
- J'ai des problèmes sévères pour me déplacer à pied
- Je suis incapable de me déplacer à pied
- ND

2 – Autonomie de la personne
- Je n'ai aucun problème pour me laver ou m'habiller tout(e) seul(e)
- J'ai des problèmes légers pour me laver ou m'habiller tout(e) seul(e)
- J'ai des problèmes modérés pour me laver ou m'habiller tout(e) seul(e)
- J'ai des problèmes sévères pour me laver ou m'habiller tout(e) seul(e)
- Je suis incapable de me laver ou de m'habiller tout(e) seul(e)
- ND

3 – Activités courantes
- Je n'ai aucun problème pour accomplir mes activités courantes
- J'ai des problèmes légers pour accomplir mes activités courantes
- J'ai des problèmes modérés pour accomplir mes activités courantes
- J'ai des problèmes sévères pour accomplir mes activités courantes
- Je suis incapable d'accomplir mes activités courantes
- ND

4 – Douleurs/ inconfort
- Je n'ai ni douleur, ni inconfort
- J'ai des douleurs ou un inconfort léger(ères)
- J'ai des douleurs ou un inconfort modéré(es)
- J'ai des douleurs ou un inconfort sévère(s)
- J'ai des douleurs ou un inconfort extrème(s)
- ND

5 – Anxiété/ dépression
- Je ne suis ni anxieux(se), ni déprimé(e)
- Je suis légèrement anxieux(se) ou déprimé(e)
- Je suis modérément anxieux(se) ou déprimé(e)
- Je suis sévèrement anxieux(se) ou déprimé(e)
- Je suis extrêmement anxieux(se) ou déprimé(e)
- ND

Etat de santé du jour : I__I__I__I/ 100

IMPEDANCEMETRIE

Masse totale du patient : I__I__I,I__I kg
Masse grasse du patient : I__I__I,I__I kg
Pourcentage en termes de masse graisseuse: I__I__I %

TRAITEMENTS

L**e patient prend-il des traitements (*traitement de fond et traitement « si besoin »*)?** : Oui / Non
Si oui, merci de bien vouloir compléter le tableau des traitements

DCI	Dose journalière	Unité
ALMOTRIPTAN		mg
ALPRAZOLAM		mg
AMITRIPTYLINE		mg
ASPIRINE		g
ATENOLOL		mg
BACLOFENE		mg
BROMAZEPAM		mg
BUPRENORPHINE		mg
BUSPIRONE		mg
CANDESARTAN		mg
CAPASAICINE		patchs par application
CARBAMAZEPINE		mg
CELECOXIB		mg
CITALOPRAM		mg
CLOBAZAM		mg
CLOMIPRAMINE		mg
CLONIDINE		mg
CLORAZEPATE		mg
CLOTIAZEPAM		mg
CODEINE		mg
DEXAMETHASONE		mg
DIAZEPAM		mg
DICLOFENAC		mg
DIHYDROCODEINE		mg
DRONABINOL		mg
DULOXETINE		mg
ELETRIPTAN		mg
ESCITALOPRAM		mg
ETORICOXIB		mg
FENTANYL		µg

FENTANYL CITRATE		µg
FLUOXETINE		mg
FROVATRIPTAN		mg
GABAPENTINE		mg
HALOPERIDOL		mg
HYDROMORPHONE		mg
IBUPROFENE		mg
INDOMETACINE		mg
KETOPROFENE		mg
LAMOTRIGINE		mg
LIDOCAINE		patchs par application
LISINOPRIL		mg
LOFLAZEPATE		mg
LOPRAZOLAM		mg
LORAZEPAN		mg
LORMETAZEPAM		mg
LOXAPINE		mg
METHADONE		mg
METHYLPREDNISOLONE		mg
METOPROLOL		mg
MIANSERINE		mg
MILNACIPRAN		mg
MIRTAZAPINE		mg
MORPHINE		mg
NADOLOL		mg
NAPROXENE		mg
NARATRIPTAN		mg
NEBIVOLOL		mg
NEFOPAM		mg
NORDAZEPAM		mg
OLANZAPINE		mg
OPIUM		mg
OXAZEPAM		mg
OXCARBAZEPINE		mg
OXYCODONE		mg
PARACETAMOL		g
PAROXETINE		mg
PHENOBARBITAL		mg
PHLOROGLUCINOL		mg
PIROXICAM		mg
PRAZEPAM		mg
PREDNISOLONE		mg
PREDNISONE		mg
PREGABALINE		mg
PROPRANOLOL		mg
RISPERIDONE		mg
RIZATRIPTAN		mg

SUMATRIPTAN		mg
THIOCOLCHICOSIDE		mg
TIMOLOL		mg
TOPIRAMATE		mg
TRAMADOL		mg
TRIMEBUTINE		mg
VALPROATE		mg
VENLAFAXINE		mg
ZOLMITRIPTAN		mg
ZOLPIDEM		mg
ZOPICLONE		mg

Patchs QUTENZA

Le patient a-t-il pris du QUTENZA SUR LES 3 MOIS PRECEDENTS LA VISITE?: Oui / Non

Si oui, I__I__I patchs par application

Visite de suivi (M6) *(+/- 15 jours)*

EVENEMENTS INDESIRABLES

➔ **Se reporter à la section « Evénements Indésirables »**
A compléter si un événement indésirable est survenu depuis la visite précédente (inclusion).

Liste des évènements indésirables à déclarer :
- Troubles fonctionnels consécutifs aux séances d'activité physiques empêchant la réalisation/ poursuite d'une activité physique
- Dysfonctionnement du système de tracking d'activité
- Faits nouveaux

DECES

➔ **Se reporter à la section « Décès »** (page de fin d'étude)

Visite de suivi (M6) *(+/- 15 jours)*

Date: I__I__I/I__I__I / I__I__I__I__I

ACTIVITE PROFESSIONNELLE

Activité professionnelle en cours : Oui/ Non/ ND

Arrêt de travail entre la visite d'inclusion et la visite de suivi M6 : Oui/ Non
Si oui, compléter le tableau ci-dessous :

**Date(s) d'arrêt**	**Date(s) de reprise associée(s)**
I__I__I/I__I__I/I__I__I__I__I	I__I__I/I__I__I/I__I__I__I__I
I__I__I/I__I__I/I__I__I__I__I	I__I__I/I__I__I/I__I__I__I__I

CONSULTATIONS MEDICALES

Consultation auprès d'un médecin généraliste depuis la dernière visite (pour un motif en lien avec la fibromyalgie ou ses conséquences)? Oui/ Non/ ND
Si oui, nombre de consultations : I__I__I

Consultation auprès d'un médecin spécialiste depuis la dernière visite (pour un motif en lien avec la fibromyalgie ou ses conséquences)? Oui/ Non/ ND
Si oui, merci de compléter le tableau ci-dessous :

**Type de spécialiste**	**Nombre de consultations**
Rhumatologue	I__I__I
Algologue	I__I__I
Psychiatre	I__I__I
Psychologue	I__I__I
Neurologue	I__I__I
Radiologue	I__I__I
Médecin du sport	I__I__I
Médecin de médecine physique, réadaptation	I__I__I
Autres, précisez : ………………………..	I__I__I
Autres, précisez : ……………………….	I__I__I

Hospitalisation depuis la dernière visite (pour un motif en lien avec la fibromyalgie ou ses conséquences) ? Oui/ Non/ ND
Si oui,

Date de début	Date de fin	Motif	Service concerné

**EVALUATION DE LA DOULEUR**
EVA : I__I__I__I/100

**MOUVEMENT EXECUTE/ IMAGINE**
Durée du premier mouvement exécuté : I__I__I__I,I__I__I secondes<br>Durée du second mouvement exécuté : I__I__I__I,I__I__I secondes<br><br>Durée du premier mouvement simulé mentalement : I__I__I__I,I__I__I secondes<br>Durée du second mouvement simulé mentalement : I__I__I__I,I__I__I secondes

**QUESTIONNAIRE FIQ-R :** Réalisé/ Non Réalisé
1 – Etes-vous capable de :<br><br>Faire les courses :<br>- Toujours<br>- La plupart du temps<br>- De temps en temps<br>- Jamais<br>- ND<br><br>Faire la lessive en machine :<br>- Toujours<br>- La plupart du temps<br>- De temps en temps<br>- Jamais<br>- ND<br><br>Faire à manger :<br>- Toujours<br>- La plupart du temps<br>- De temps en temps<br>- Jamais<br>- ND<br><br>Faire la vaisselle à la main :<br>- Toujours<br>- La plupart du temps<br>- De temps en temps<br>- Jamais<br>- ND

Passer l'aspirateur :
- Toujours (
- La plupart du temps
- De temps en temps
- Jamais
- ND

Faire les lits :
- Toujours
- La plupart du temps
- De temps en temps
- Jamais
- ND

Marcher plusieurs centaines de mètres :
- Toujours
- La plupart du temps
- De temps en temps
- Jamais
- ND

Aller voir des amis ou de la famille :
- Toujours
- La plupart du temps
- De temps en temps
- Jamais
- ND

Faire du jardinage :
- Toujours
- La plupart du temps
- De temps en temps
- Jamais
- ND

Conduire une voiture :
- Toujours)
- La plupart du temps
- De temps en temps
- Jamais)
- ND

Au cours des 7 derniers jours :

2 – Combien de jour vous êtes-vous senti(e) bien ? I__I

3 – Combien de journées de travail avez-vous manqué à cause de votre fibromyalgie ? I__I

4 – Les jours où vous avez travaillé, les douleurs ou d'autres problèmes liés à votre fibromyalgie vous ont-ils gêné dans votre travail ? I__I__I,I__I

5 – Avez-vous des douleurs ? I__I__I,I__I

6 – Avez-vous été fatigué(e) ? I__I__I,I__I

7 – Comment vous êtes-vous senti(e) le matin au réveil ? I__I__I,I__I

8 – Vous êtes-vous senti(e) raide ? I__I__I,I__I

9 – Vous êtes-vous senti(e) tendu(e) ou inquiet(e) ? I__I__I,I__I (

10 – Vous êtes-vous senti(e) déprimé(e) ? I__I__I,I__I

Score global: ……../100

QUESTIONNAIRE DN4 : Réalisé/ Non réalisé

1 – La douleur présente-t-elle une ou plusieurs des caractéristiques suivantes ?
- Brulure : Oui/ Non/ ND
- Sensation de froid douloureux: Oui/ Non/ ND
- Décharges électriques : Oui/ Non/ ND

2 – La douleur est-elle associée dans la même région à un ou plusieurs des symptômes suivants ?
- Fourmillements : Oui/ Non/ ND
- Picotements: Oui/ Non/ ND
- Engourdissements : Oui/ Non/ ND
- Démangeaisons : Oui/ Non/ ND

3 – La douleur est-elle localisée dans un territoire où l'examen met en évidence :
- Hypoesthésie au tact ? Oui/ Non/ ND
- Hypoesthésie à la piqûre ? Oui/ Non/ ND

4 – La douleur est-elle provoquée ou augmentée par :
- Le frottement ? Oui/ Non/ ND

Score du patient : …../10

QUESTIONNAIRE HAD : Réalisé/ Non réalisé

1 – Je me sens tendu(e) ou énervé(e) :
- La plupart du temps
- Souvent
- De temps en temps
- Jamais
- ND

2 – Je prends plaisir aux mêmes choses qu'autrefois :
- Oui, tout autant
- Pas autant
- Un peu seulement
- Presque plus
- ND

3 – J'ai une sensation de peur comme si quelque chose d'horrible allait m'arriver :
- Oui, très nettement
- Oui, mais ce n'est pas trop grave
- Un peu, mais cela ne m'inquiète pas
- Pas du tout
- ND

4 – Je ris facilement et vois le bon côté des choses :
- Autant que par le passé
- Plus autant qu'avant
- Vraiment moins qu'avant
- Plus du tout
- ND

5 – Je me fais du souci :
- Très souvent
- Assez souvent
- Occasionnellement
- Très occasionnellement
- ND

6 – Je suis de bonne humeur :
- Jamais
- Rarement
- Assez souvent
- La plupart du temps
- ND

7 – Je peux rester tranquillement assis(se) à ne rien faire et me sentir décontracté(e) :
- Oui, quoi qu'il arrive
- Oui, en général
- Rarement
- Jamais
- ND

8 – J'ai l'impression de fonctionner au ralenti :
- Presque toujours
- Très souvent
- Parfois
- Jamais
- ND

9 – J'éprouve des sensations de peur et j'ai l'estomac noué :
- Jamais
- Parfois
- Assez souvent
- Très souvent
- ND

10 – Je ne m'intéresse plus à mon apparence :
- Plus du tout
- Je n'y accorde pas autant d'attention que je devrais
- Il se peut que je n'y fasse plus autant attention
- J'y prête autant d'attention que par le passé
- ND

11 – J'ai la bougeotte et n'arrive pas à tenir en place:
- Oui, c'est tout à fait le cas
- Un peu
- Pas tellement
- Pas du tout
- ND

12 – Je me réjouis d'avance à l'idée de faire certaines choses :
- Autant qu'avant
- Un peu moins qu'avant
- Bien moins qu'avant
- Presque jamais
- ND

13 – J'éprouve des sensations soudaines de panique:
- Vraiment très souvent
- Assez souvent
- Pas très souvent
- Jamais
- ND

14 – Je peux prendre plaisir à un bon livre ou à une bonne émission de radio ou de télévision :
- Souvent
- Parfois
- Rarement
- Très rarement
- ND

Score en lien avec l'anxiété : …../ 21
Score en lien avec la dimension dépressive : ….. / 21

QUESTIONNAIRE WPI : Réalisé/ Non réalisé

Nombre de zones pour lesquelles le patient a ressenti de la douleur la semaine précédente : I__I__I/19

ECHELLE DE SEVERITE DES SYMPTOMES

Intensité des symptômes (au cours de la semaine passée): Réalisé/ Non Réalisé

4- Fatigue
- Pas de problème
- Problèmes légers ou modérés, généralement modérés ou intermittents
- Problèmes modérés, considérables, souvent présents et/ou à niveau modéré
- Problèmes importants, persistants, continus, handicapants au quotidien
- ND

5- Somnolence
- Pas de problème
- Problèmes légers ou modérés, généralement modérés ou intermittents
- Problèmes modérés, considérables, souvent présents et/ou à niveau modéré
- Problèmes importants, persistants, continus, handicapants au quotidien
- ND

6- Symptômes cognitifs
- Pas de problème
- Problèmes légers ou modérés, généralement modérés ou intermittents
- Problèmes modérés, considérables, souvent présents et/ou à niveau modéré
- Problèmes importants, persistants, continus, handicapants au quotidien
- ND

Score : ………./9

Nombre de symptômes associés (au cours de la semaine passée) : Réalisé/ Non Réalisé

☐ Douleur musculaire	☐ Syndrome intestin irritable	☐ Fatigue
☐ Problème mémoire, concentration	☐ Céphalées	☐ Douleur/ crampes abdominales
☐ Faiblesse musculaire	☐ Engourdissement/ picotement	☐ Vertiges
☐ Insomnie	☐ Dépression	☐ Nervosité
☐ Constipation	☐ Douleur abdominale haute	☐ Nausée
☐ Douleur thoracique	☐ Diarrhée	☐ Bouche sèche
☐ Vision floue	☐ Fièvre	☐ Démangeaisons
☐ Sifflement respiratoire	☐ Syndrome de Raynaud	☐ Urticaire
☐ Acouphènes	☐ Vomissements	☐ Brûlure thoracique
☐ Aphtes buccaux	☐ Perte ou modification du goût	☐ Crise d'épilepsie
☐ Yeux secs	☐ Oppression respiratoire	☐ Perte d'appétit

☐ Eruption cutanée	☐ Hypersensibilité au soleil	☐ Troubles de l'audition
☐ Ecchymoses faciles	☐ Perte de cheveux	☐ Mictions fréquentes
☐ Douleurs à la miction	☐ Spasmes de la vessie	

QUESTIONNAIRE DE TAMPA : Réalisé/ Non réalisé

18- J'ai peur de me blesser si je fais de l'activité physique :
- Fortement en désaccord
- Quelque peu en désaccord
- Quelque peu en accord
- Fortement en accord
- ND

19- Ma douleur ne ferait qu'intensifier si j'essayais de la vaincre :
- Fortement en désaccord
- Quelque peu en désaccord
- Quelque peu en accord
- Fortement en accord
- ND

20- Mon corps me dit que quelque chose ne va vraiment pas :
- Fortement en désaccord
- Quelque peu en désaccord
- Quelque peu en accord
- Fortement en accord
- ND

21- Si je faisais de l'activité physique, ma douleur serait probablement soulagée:
- Fortement en désaccord
- Quelque peu en désaccord
- Quelque peu en accord
- Fortement en accord
- ND

22- Les gens ne prennent pas mon état de santé au sérieux :
- Fortement en désaccord
- Quelque peu en désaccord
- Quelque peu en accord
- Fortement en accord
- ND

23- Mon accident a mis mon corps en danger pour le reste de mes jours :
- Fortement en désaccord
- Quelque peu en désaccord
- Quelque peu en accord
- Fortement en accord
- ND

24- La douleur signifie toujours que je me suis blessé(e) :
- Fortement en désaccord
- Quelque peu en désaccord
- Quelque peu en accord
- Fortement en accord
- ND

25- Même si quelque chose aggrave ma douleur, cela ne veut pas dire que c'est dangereux :
- Fortement en désaccord
- Quelque peu en désaccord
- Quelque peu en accord
- Fortement en accord
- ND

26- J'ai peur de me blesser accidentellement :
- Fortement en désaccord
- Quelque peu en désaccord
- Quelque peu en accord
- Fortement en accord
- ND

27- La meilleure façon d'empêcher que ma douleur s'aggrave est de m'assurer de ne pas faire des mouvements inutiles :
- Fortement en désaccord
- Quelque peu en désaccord
- Quelque peu en accord
- Fortement en accord
- ND

28- Je n'aurais pas tant que douleurs s'il ne se passait pas quelque chose de grave dans mon corps :
- Fortement en désaccord
- Quelque peu en désaccord
- Quelque peu en accord
- Fortement en accord
- ND

29- Bien que ma condition soit pénible, je serais mieux si j'étais physiquement actif(ve) :
- Fortement en désaccord
- Quelque peu en désaccord
- Quelque peu en accord
- Fortement en accord
- ND

30- La douleur m'indique quand arrêter de faires des activités physiques pour que je ne me blesse pas :
- Fortement en désaccord
- Quelque peu en désaccord
- Quelque peu en accord
- Fortement en accord
- ND

31- Il n'est pas prudent qu'une personne avec un état de santé comme le mien soit physiquement

active :
- Fortement en désaccord
- Quelque peu en désaccord
- Quelque peu en accord
- Fortement en accord
- ND

32- Je ne peux pas faire tout ce qu'une personne normale peut faire parce que j'ai plus de risque de me blesser :
- Fortement en désaccord
- Quelque peu en désaccord
- Quelque peu en accord
- Fortement en accord
- ND

33- Bien qu'il y ait quelque chose qui me cause beaucoup de douleurs, je ne pense pas que ce soit vraiment grave :
- Fortement en désaccord
- Quelque peu en désaccord
- Quelque peu en accord
- Fortement en accord
- ND

34- Personne ne devrait être obligé de faire des exercices lorsqu'il (elle) ressent de la douleur :
- Fortement en désaccord
- Quelque peu en désaccord
- Quelque peu en accord
- Fortement en accord
- ND

Score : ……/68

EQ-5D-5L : Réalisé/ Non Réalisé

2- Mobilité
- Je n'ai aucun problème pour me déplacer à pied
- J'ai des problèmes légers pour me déplacer à pied
- J'ai des problèmes modérés pour me déplacer à pied
- J'ai des problèmes sévères pour me déplacer à pied
- Je suis incapable de me déplacer à pied
- ND

2 – Autonomie de la personne
- Je n'ai aucun problème pour me laver ou m'habiller tout(e) seul(e)
- J'ai des problèmes légers pour me laver ou m'habiller tout(e) seul(e)
- J'ai des problèmes modérés pour me laver ou m'habiller tout(e) seul(e)
- J'ai des problèmes sévères pour me laver ou m'habiller tout(e) seul(e)
- Je suis incapable de me laver ou de m'habiller tout(e) seul(e)
- ND

3 – Activités courantes

- Je n’ai aucun problème pour accomplir mes activités courantes
- J’ai des problèmes légers pour accomplir mes activités courantes
- J’ai des problèmes modérés pour accomplir mes activités courantes
- J’ai des problèmes sévères pour accomplir mes activités courantes
- Je suis incapable d’accomplir mes activités courantes
- ND

4 – Douleurs/ inconfort

- Je n’ai ni douleur, ni inconfort
- J’ai des douleurs ou un inconfort léger(ères)
- J’ai des douleurs ou un inconfort modéré(es)
- J’ai des douleurs ou un inconfort sévère(s)
- J’ai des douleurs ou un inconfort extrème(s)
- ND

5 – Anxiété/ dépression

- Je ne suis ni anxieux(se), ni déprimé(e)
- Je suis légèrement anxieux(se) ou déprimé(e)
- Je suis modérément anxieux(se) ou déprimé(e)
- Je suis sévèrement anxieux(se) ou déprimé(e)
- Je suis extrêmement anxieux(se) ou déprimé(e)
- ND

Etat de santé du jour : I__I__I__I/ 100

IMPEDANCEMETRIE

Masse totale du patient : I__I__I,I__I kg
Masse grasse du patient : I__I__I,I__I kg
Pourcentage en termes de masse graisseuse: I__I__I %

TRAITEMENTS

Le patient prend-il des traitements (*traitement de fond et traitement « si besoin »*)?: Oui / Non
Si oui, merci de bien vouloir compléter le tableau des traitements

DCI	Dose journalière	Unité
ALMOTRIPTAN		mg
ALPRAZOLAM		mg
AMITRIPTYLINE		mg
ASPIRINE		g
ATENOLOL		mg
BACLOFENE		mg
BROMAZEPAM		mg
BUPRENORPHINE		mg
BUSPIRONE		mg
CANDESARTAN		mg
CAPASAICINE		patchs par application
CARBAMAZEPINE		mg
CELECOXIB		mg
CITALOPRAM		mg
CLOBAZAM		mg
CLOMIPRAMINE		mg
CLONIDINE		mg
CLORAZEPATE		mg
CLOTIAZEPAM		mg
CODEINE		mg
DEXAMETHASONE		mg
DIAZEPAM		mg
DICLOFENAC		mg
DIHYDROCODEINE		mg
DRONABINOL		mg
DULOXETINE		mg
ELETRIPTAN		mg
ESCITALOPRAM		mg
ETORICOXIB		mg
FENTANYL		µg
FENTANYL CITRATE		µg
FLUOXETINE		mg
FROVATRIPTAN		mg
GABAPENTINE		mg
HALOPERIDOL		mg
HYDROMORPHONE		mg
IBUPROFENE		mg
INDOMETACINE		mg

KETOPROFENE		mg
LAMOTRIGINE		mg
LIDOCAINE		patchs par application
LISINOPRIL		mg
LOFLAZEPATE		mg
LOPRAZOLAM		mg
LORAZEPAN		mg
LORMETAZEPAM		mg
LOXAPINE		mg
METHADONE		mg
METHYLPREDNISOLONE		mg
METOPROLOL		mg
MIANSERINE		mg
MILNACIPRAN		mg
MIRTAZAPINE		mg
MORPHINE		mg
NADOLOL		mg
NAPROXENE		mg
NARATRIPTAN		mg
NEBIVOLOL		mg
NEFOPAM		mg
NORDAZEPAM		mg
OLANZAPINE		mg
OPIUM		mg
OXAZEPAM		mg
OXCARBAZEPINE		mg
OXYCODONE		mg
PARACETAMOL		g
PAROXETINE		mg
PHENOBARBITAL		mg
PHLOROGLUCINOL		mg
PIROXICAM		mg
PRAZEPAM		mg
PREDNISOLONE		mg
PREDNISONE		mg
PREGABALINE		mg
PROPRANOLOL		mg
RISPERIDONE		mg
RIZATRIPTAN		mg
SUMATRIPTAN		mg
THIOCOLCHICOSIDE		mg
TIMOLOL		mg
TOPIRAMATE		mg
TRAMADOL		mg
TRIMEBUTINE		mg
VALPROATE		mg
VENLAFAXINE		mg

ZOLMITRIPTAN		mg
ZOLPIDEM		mg
ZOPICLONE		mg

Patchs QUTENZA

Le patient a-t-il pris du QUTENZA SUR LES 3 MOIS PRECEDENTS LA VISITE? : Oui / Non

Si oui, I__I__I patchs par application

Visite de suivi (M12) *(+/- 15 jours)*

EVENEMENTS INDESIRABLES

➔ **Se reporter à la section « Evénements Indésirables »**
A compléter si un événement indésirable est survenu depuis la visite précédente (M6).

Liste des évènements indésirables à déclarer :
- Troubles fonctionnels consécutifs aux séances d'activité physique empêchant la réalisation/ poursuite d'une activité physique
- Dysfonctionnement du système de tracking
- Faits nouveaux

DECES

➔ **Se reporter à la section « Décès »** (page de fin d'étude)

Visite de suivi (M12) *(+/- 15 jours)*

Date: I__I__I/I__I__I / I__I__I__I__I

ACTIVITE PROFESSIONNELLE

Activité professionnelle en cours : Oui/ Non/ ND

Arrêt de travail entre la visite de suivi M6 et la visite de suivi M12 : Oui/ Non
Si oui, compléter le tableau ci-dessous :

Date(s) d'arrêt	Date(s) de reprise associée(s)
I__I__I/I__I__I/I__I__I__I__I	I__I__I/I__I__I/I__I__I__I__I
I__I__I/I__I__I/I__I__I__I__I	I__I__I/I__I__I/I__I__I__I__I

CONSULTATIONS MEDICALES

Consultation auprès d'un médecin généraliste depuis la dernière visite (pour un motif en lien avec la fibromyalgie ou ses conséquences)? Oui/ Non/ ND

Si oui, nombre de consultations : I__I__I

Consultation auprès d'un médecin spécialiste depuis la dernière visite (pour un motif en lien avec la fibromyalgie ou ses conséquences)? Oui/ Non/ ND

Si oui, merci de compléter le tableau ci-dessous :

Type de spécialiste	Nombre de consultations
Rhumatologue	I__I__I
Algologue	I__I__I
Psychiatre	I__I__I
Psychologue	I__I__I
Neurologue	I__I__I
Radiologue	I__I__I
Médecin du sport	I__I__I
Médecin de médecine physique, réadaptation	I__I__I
Autres, précisez : ………………………..	I__I__I
Autres, précisez : ……………………….	I__I__I

Hospitalisation depuis la dernière visite (pour un motif en lien avec la fibromyalgie ou ses conséquences) ? Oui/ Non/ ND

Si oui,

Date de début	Date de fin	Motif	Service concerné

EVALUATION DE LA DOULEUR
EVA : I__I__I__I/100

MOUVEMENT EXECUTE/ IMAGINE
Durée du premier mouvement exécuté : I__I__I__I,I__I__I secondes<br>Durée du second mouvement exécuté : I__I__I__I,I__I__I secondes<br><br>Durée du premier mouvement simulé mentalement : I__I__I__I,I__I__I secondes<br>Durée du second mouvement simulé mentalement : I__I__I__I,I__I__I secondes

QUESTIONNAIRE FIQ-R : Réalisé/ Non Réalisé

1 – Etes-vous capable de :

Faire les courses :
- Toujours
- La plupart du temps
- De temps en temps
- Jamais
- ND

Faire la lessive en machine :
- Toujours
- La plupart du temps
- De temps en temps
- Jamais
- ND

Faire à manger :
- Toujours
- La plupart du temps
- De temps en temps
- Jamais
- ND

Faire la vaisselle à la main :
- Toujours
- La plupart du temps
- De temps en temps
- Jamais
- ND

Passer l'aspirateur :
- Toujours (
- La plupart du temps
- De temps en temps
- Jamais
- ND

Faire les lits :
- Toujours
- La plupart du temps
- De temps en temps
- Jamais
- ND

Marcher plusieurs centaines de mètres :
- Toujours
- La plupart du temps
- De temps en temps
- Jamais
- ND

Aller voir des amis ou de la famille :
- Toujours
- La plupart du temps
- De temps en temps
- Jamais
- ND

Faire du jardinage :
- Toujours
- La plupart du temps
- De temps en temps
- Jamais
- ND

Conduire une voiture :
- Toujours)
- La plupart du temps
- De temps en temps
- Jamais)
- ND

Au cours des 7 derniers jours :

2 – Combien de jour vous êtes-vous senti(e) bien ? I__I

3 – Combien de journées de travail avez-vous manqué à cause de votre fibromyalgie ? I__I

4 – Les jours où vous avez travaillé, les douleurs ou d'autres problèmes liés à votre fibromyalgie vous ont-ils gêné dans votre travail ? I__I__I,I__I

5 – Avez-vous des douleurs ? I__I__I,I__I

6 – Avez-vous été fatigué(e) ? I__I__I,I__I

7 – Comment vous êtes-vous senti(e) le matin au réveil ? I__I__I,I__I

8 – Vous êtes-vous senti(e) raide ? I__I__I,I__I

9 – Vous êtes-vous senti(e) tendu(e) ou inquiet(e) ? I__I__I,I__I (

10 – Vous êtes-vous senti(e) déprimé(e) ? I__I__I,I__I

Score global: ……../100

QUESTIONNAIRE DN4 : Réalisé/ Non réalisé

1 – La douleur présente-t-elle une ou plusieurs des caractéristiques suivantes ?
- Brulure : Oui/ Non/ ND
- Sensation de froid douloureux: Oui/ Non/ ND
- Décharges électriques : Oui/ Non/ ND

2 – La douleur est-elle associée dans la même région à un ou plusieurs des symptômes suivants ?
- Fourmillements : Oui/ Non/ ND
- Picotements: Oui/ Non/ ND
- Engourdissements : Oui/ Non/ ND
- Démangeaisons : Oui/ Non/ ND

3 – La douleur est-elle localisée dans un territoire où l'examen met en évidence :
- Hypoesthésie au tact ? Oui/ Non/ ND
- Hypoesthésie à la piqûre ? Oui/ Non/ ND

4 – La douleur est-elle provoquée ou augmentée par :
- Le frottement ? Oui/ Non/ ND

QUESTIONNAIRE HAD : Réalisé/ Non réalisé

1 – Je me sens tendu(e) ou énervé(e) :
- La plupart du temps
- Souvent
- De temps en temps
- Jamais
- ND

2 – Je prends plaisir aux mêmes choses qu'autrefois :
- Oui, tout autant
- Pas autant
- Un peu seulement
- Presque plus
- ND

3 – J'ai une sensation de peur comme si quelque chose d'horrible allait m'arriver :
- Oui, très nettement
- Oui, mais ce n'est pas trop grave
- Un peu, mais cela ne m'inquiète pas
- Pas du tout
- ND

4 – Je ris facilement et vois le bon côté des choses :
- Autant que par le passé
- Plus autant qu'avant
- Vraiment moins qu'avant
- Plus du tout
- ND

5 – Je me fais du souci :
- Très souvent
- Assez souvent
- Occasionnellement
- Très occasionnellement
- ND

6 – Je suis de bonne humeur :
- Jamais
- Rarement
- Assez souvent
- La plupart du temps
- ND

7 – Je peux rester tranquillement assis(se) à ne rien faire et me sentir décontracté(e) :
- Oui, quoi qu'il arrive
- Oui, en général
- Rarement
- Jamais
- ND

8 – J'ai l'impression de fonctionner au ralenti :
- Presque toujours
- Très souvent
- Parfois
- Jamais
- ND

9 – J'éprouve des sensations de peur et j'ai l'estomac noué :
- Jamais
- Parfois
- Assez souvent
- Très souvent
- ND

10 – Je ne m'intéresse plus à mon apparence :
- Plus du tout
- Je n'y accorde pas autant d'attention que je devrais
- Il se peut que je n'y fasse plus autant attention
- J'y prête autant d'attention que par le passé
- ND

11 – J'ai la bougeotte et n'arrive pas à tenir en place:
- Oui, c'est tout à fait le cas
- Un peu
- Pas tellement
- Pas du tout
- ND

12 – Je me réjouis d'avance à l'idée de faire certaines choses :
- Autant qu'avant
- Un peu moins qu'avant
- Bien moins qu'avant
- Presque jamais
- ND

13 – J'éprouve des sensations soudaines de panique:
- Vraiment très souvent
- Assez souvent
- Pas très souvent
- Jamais
- ND

14 – Je peux prendre plaisir à un bon livre ou à une bonne émission de radio ou de télévision :
- Souvent
- Parfois
- Rarement
- Très rarement
- ND

Score en lien avec l'anxiété : …../ 21
Score en lien avec la dimension dépressive : ….. / 21

**QUESTIONNAIRE WPI :** Réalisé/ Non réalisé
Nombre de zones pour lesquelles le patient a ressenti de la douleur la semaine précédente : I__I__I/19

ECHELLE DE SEVERITE DES SYMPTOMES

Intensité des symptômes (au cours de la semaine passée): Réalisé/ Non Réalisé

7- Fatigue
- Pas de problème
- Problèmes légers ou modérés, généralement modérés ou intermittents
- Problèmes modérés, considérables, souvent présents et/ou à niveau modéré
- Problèmes importants, persistants, continus, handicapants au quotidien
- ND

8- Somnolence
- Pas de problème
- Problèmes légers ou modérés, généralement modérés ou intermittents
- Problèmes modérés, considérables, souvent présents et/ou à niveau modéré
- Problèmes importants, persistants, continus, handicapants au quotidien
- ND

9- Symptômes cognitifs
- Pas de problème
- Problèmes légers ou modérés, généralement modérés ou intermittents
- Problèmes modérés, considérables, souvent présents et/ou à niveau modéré
- Problèmes importants, persistants, continus, handicapants au quotidien
- ND

Score : ………./9

Nombre de symptômes associés (au cours de la semaine passée) : Réalisé/ Non Réalisé

☐ Douleur musculaire	☐Syndrome intestin irritable	☐Fatigue
☐Problème mémoire, concentration	☐Céphalées	☐Douleur/ crampes abdominales
☐Faiblesse musculaire	☐Engourdissement/ picotement	☐Vertiges
☐Insomnie	☐Dépression	☐Nervosité
☐Constipation	☐Douleur abdominale haute	☐Nausée
☐Douleur thoracique	☐Diarrhée	☐Bouche sèche
☐Vision floue	☐Fièvre	☐Démangeaisons
☐Sifflement respiratoire	☐Syndrome de Raynaud	☐Urticaire
☐Acouphènes	☐Vomissements	☐Brûlure thoracique
☐Aphtes buccaux	☐Perte ou modification du goût	☐Crise d'épilepsie
☐Yeux secs	☐Oppression respiratoire	☐Perte d'appétit
☐Eruption cutanée	☐Hypersensibilité au soleil	☐Troubles de l'audition
☐Ecchymoses faciles	☐Perte de cheveux	☐Mictions fréquentes

☐Douleurs à la miction	☐Spasmes de la vessie	

Score : ……………/3

Score total : ……………/12

QUESTIONNAIRE DE TAMPA : Réalisé/ Non réalisé

35- J'ai peur de me blesser si je fais de l'activité physique :
- Fortement en désaccord
- Quelque peu en désaccord
- Quelque peu en accord
- Fortement en accord
- ND

36- Ma douleur ne ferait qu'intensifier si j'essayais de la vaincre :
- Fortement en désaccord
- Quelque peu en désaccord
- Quelque peu en accord
- Fortement en accord
- ND

37- Mon corps me dit que quelque chose ne va vraiment pas :
- Fortement en désaccord
- Quelque peu en désaccord
- Quelque peu en accord
- Fortement en accord
- ND

38- Si je faisais de l'activité physique, ma douleur serait probablement soulagée:
- Fortement en désaccord
- Quelque peu en désaccord
- Quelque peu en accord
- Fortement en accord
- ND

39- Les gens ne prennent pas mon état de santé au sérieux :
- Fortement en désaccord
- Quelque peu en désaccord
- Quelque peu en accord
- Fortement en accord
- ND

40- Mon accident a mis mon corps en danger pour le reste de mes jours :
- Fortement en désaccord
- Quelque peu en désaccord
- Quelque peu en accord
- Fortement en accord
- ND

41- La douleur signifie toujours que je me suis blessé(e) :
- Fortement en désaccord
- Quelque peu en désaccord
- Quelque peu en accord
- Fortement en accord
- ND

42- Même si quelque chose aggrave ma douleur, cela ne veut pas dire que c'est dangereux :
- Fortement en désaccord
- Quelque peu en désaccord
- Quelque peu en accord
- Fortement en accord
- ND

43- J'ai peur de me blesser accidentellement :
- Fortement en désaccord
- Quelque peu en désaccord
- Quelque peu en accord
- Fortement en accord
- ND

44- La meilleure façon d'empêcher que ma douleur s'aggrave est de m'assurer de ne pas faire des mouvements inutiles :
- Fortement en désaccord
- Quelque peu en désaccord
- Quelque peu en accord
- Fortement en accord
- ND

45- Je n'aurais pas tant que douleurs s'il ne se passait pas quelque chose de grave dans mon corps :
- Fortement en désaccord
- Quelque peu en désaccord
- Quelque peu en accord
- Fortement en accord
- ND

46- Bien que ma condition soit pénible, je serais mieux si j'étais physiquement actif(ve) :
- Fortement en désaccord
- Quelque peu en désaccord
- Quelque peu en accord
- Fortement en accord
- ND

47- La douleur m'indique quand arrêter de faires des activités physiques pour que je ne me blesse pas :
- Fortement en désaccord
- Quelque peu en désaccord
- Quelque peu en accord
- Fortement en accord
- ND

48- Il n'est pas prudent qu'une personne avec un état de santé comme le mien soit physiquement active :

- Fortement en désaccord
- Quelque peu en désaccord
- Quelque peu en accord
- Fortement en accord
- ND

49- Je ne peux pas faire tout ce qu'une personne normale peut faire parce que j'ai plus de risque de me blesser :
- Fortement en désaccord
- Quelque peu en désaccord
- Quelque peu en accord
- Fortement en accord
- ND

50- Bien qu'il y ait quelque chose qui me cause beaucoup de douleurs, je ne pense pas que ce soit vraiment grave :
- Fortement en désaccord
- Quelque peu en désaccord
- Quelque peu en accord
- Fortement en accord
- ND

51- Personne ne devrait être obligé de faire des exercices lorsqu'il (elle) ressent de la douleur :
- Fortement en désaccord
- Quelque peu en désaccord
- Quelque peu en accord
- Fortement en accord
- ND

Score : ……/68

EQ-5D-5L : Réalisé/ Non Réalisé

3- Mobilité
- Je n'ai aucun problème pour me déplacer à pied
- J'ai des problèmes légers pour me déplacer à pied
- J'ai des problèmes modérés pour me déplacer à pied
- J'ai des problèmes sévères pour me déplacer à pied
- Je suis incapable de me déplacer à pied
- ND

2 – Autonomie de la personne
- Je n'ai aucun problème pour me laver ou m'habiller tout(e) seul(e)
- J'ai des problèmes légers pour me laver ou m'habiller tout(e) seul(e)
- J'ai des problèmes modérés pour me laver ou m'habiller tout(e) seul(e)
- J'ai des problèmes sévères pour me laver ou m'habiller tout(e) seul(e)
- Je suis incapable de me laver ou de m'habiller tout(e) seul(e)
- ND

3 – Activités courantes

- Je n'ai aucun problème pour accomplir mes activités courantes
- J'ai des problèmes légers pour accomplir mes activités courantes
- J'ai des problèmes modérés pour accomplir mes activités courantes
- J'ai des problèmes sévères pour accomplir mes activités courantes
- Je suis incapable d'accomplir mes activités courantes
- ND

4 – Douleurs/ inconfort
- Je n'ai ni douleur, ni inconfort
- J'ai des douleurs ou un inconfort léger(ères)
- J'ai des douleurs ou un inconfort modéré(es)
- J'ai des douleurs ou un inconfort sévère(s)
- J'ai des douleurs ou un inconfort extrème(s)
- ND

5 – Anxiété/ dépression
- Je ne suis ni anxieux(se), ni déprimé(e)
- Je suis légèrement anxieux(se) ou déprimé(e)
- Je suis modérément anxieux(se) ou déprimé(e)
- Je suis sévèrement anxieux(se) ou déprimé(e)
- Je suis extrêmement anxieux(se) ou déprimé(e)
- ND

IMPEDANCEMETRIE

Masse totale du patient : I__I__I,I__I kg
Masse grasse du patient : I__I__I,I__I kg
Pourcentage en termes de masse graisseuse: I__I__I %

ACTIVITE PHYSIQUE

Continuité de l'activité physique après M6: Oui/ Non/ ND

TRAITEMENTS

Le patient prend-il des traitements (*traitement de fond et traitement « si besoin »*)? : Oui / Non
Si oui, merci de bien vouloir compléter le tableau des traitements

DCI	Dose journalière	Unité
ALMOTRIPTAN		mg
ALPRAZOLAM		mg
AMITRIPTYLINE		mg
ASPIRINE		g
ATENOLOL		mg
BACLOFENE		mg
BROMAZEPAM		mg
BUPRENORPHINE		mg
BUSPIRONE		mg
CANDESARTAN		mg
CAPASAICINE		patchs par application
CARBAMAZEPINE		mg
CELECOXIB		mg
CITALOPRAM		mg
CLOBAZAM		mg
CLOMIPRAMINE		mg
CLONIDINE		mg
CLORAZEPATE		mg
CLOTIAZEPAM		mg
CODEINE		mg
DEXAMETHASONE		mg
DIAZEPAM		mg
DICLOFENAC		mg
DIHYDROCODEINE		mg
DRONABINOL		mg
DULOXETINE		mg
ELETRIPTAN		mg
ESCITALOPRAM		mg
ETORICOXIB		mg
FENTANYL		µg
FENTANYL CITRATE		µg
FLUOXETINE		mg
FROVATRIPTAN		mg
GABAPENTINE		mg
HALOPERIDOL		mg
HYDROMORPHONE		mg
IBUPROFENE		mg
INDOMETACINE		mg
KETOPROFENE		mg

LAMOTRIGINE		mg
LIDOCAINE		patchs par application
LISINOPRIL		mg
LOFLAZEPATE		mg
LOPRAZOLAM		mg
LORAZEPAN		mg
LORMETAZEPAM		mg
LOXAPINE		mg
METHADONE		mg
METHYLPREDNISOLONE		mg
METOPROLOL		mg
MIANSERINE		mg
MILNACIPRAN		mg
MIRTAZAPINE		mg
MORPHINE		mg
NADOLOL		mg
NAPROXENE		mg
NARATRIPTAN		mg
NEBIVOLOL		mg
NEFOPAM		mg
NORDAZEPAM		mg
OLANZAPINE		mg
OPIUM		mg
OXAZEPAM		mg
OXCARBAZEPINE		mg
OXYCODONE		mg
PARACETAMOL		g
PAROXETINE		mg
PHENOBARBITAL		mg
PHLOROGLUCINOL		mg
PIROXICAM		mg
PRAZEPAM		mg
PREDNISOLONE		mg
PREDNISONE		mg
PREGABALINE		mg
PROPRANOLOL		mg
RISPERIDONE		mg
RIZATRIPTAN		mg
SUMATRIPTAN		mg
THIOCOLCHICOSIDE		mg
TIMOLOL		mg
TOPIRAMATE		mg
TRAMADOL		mg
TRIMEBUTINE		mg
VALPROATE		mg
VENLAFAXINE		mg
ZOLMITRIPTAN		mg

ZOLPIDEM		mg
ZOPICLONE		mg

Patchs QUTENZA

L**e patient a-t-il pris du QUTENZA <u>SUR LES 3 MOIS PRECEDENTS LA VISITE</u>?** : Oui / Non

Si oui, I__I__I patchs par application

FREQUENCE CARDIAQUE MAXIMALE THEORIQUE

Durée dans chaque intervalle de la fréquence cardiaque théorique :

**Durées** / **Semaines**	Entre 50 et 60% de la FCM	Durée entre 60 et 70% de la FCM	Durée entre 70 et 80% de la FCM	Durée entre 80 et 90% de la FCM	Nombre de séances
S1					
S2					
S3					
S4					
S5					
S6					
S7					
S8					
S9					
S10					
S11					
S12					
S13					
S14					
S15					

**EVENEMENTS INDESIRABLES (EvI)** *(cf. EI d'intérêts listés dans le protocole)* **:** Oui/ Non				
**N°**	**Nature de l'événement**	**Catégorie d'évènement**	**Date de début**	**Date de résolution**
1	__________		I_I_I / I_I_I / I_I_I_I_I	I_I_I / I_I_I / I_I_I_I_I
2	__________		I_I_I / I_I_I / I_I_I_I_I	I_I_I / I_I_I / I_I_I_I_I
3	__________		I_I_I / I_I_I / I_I_I_I_I	I_I_I / I_I_I / I_I_I_I_I
4	__________		I_I_I / I_I_I / I_I_I_I_I	I_I_I / I_I_I / I_I_I_I_I

Signalement d'un début de grossesse : Oui/ Non
Si oui, date de début de grossesse: I__I__I/I__I__I/I__I__I__I__I

*Catégorie d'évènement : 1= Troubles fonctionnels consécutifs aux séances empêchant la réalisation/ poursuite d'une activité physique ; 2= Dysfonctionnement du système de tracking d'activité ; 3= Faits nouveaux

STATUT DU PATIENT EN FIN D'ETUDE

La date du dernier jour de participation à l'étude correspond-elle à la date finale théorique de fin d'étude : Oui/ Non

Date du dernier jour de participation à l'étude: I__I__I / I__I__I / I__I__I__I__I

En cas de sortie prématurée d'étude, préciser la raison:

- ☐ Retrait de consentement[(1)]
- ☐ Perdu de vue
- ☐ Décès[(2)]
- ☐ Déviation au protocole empêchant la poursuite de l'étude[(3)]

[(1)] *En cas de retrait de consentement, compléter la section « Retrait de consentement ».*

[(2)] *En cas de décès, compléter la section « Décès ».*

[(3)] *En cas de déviation au protocole, compléter le tableau des déviations.*

RETRAIT DE CONSENTEMENT

Si retrait de consentement,

- Date du retrait de consentement : I__I__I / I__I__I / I__I__I__I__I
- Le patient s'oppose-t-il à l'analyse de ses données? Oui/ Non

DEVIATIONS

Déviation(s) au protocole: Oui/ Non

(Il peut s'agir, à titre d'exemples, de déviation liée au non-respect d'un critère d'inclusion, d'un non-respect du bras de randomisation, d'une visite non réalisée, etc…)

Si oui, préciser le type de déviation et la décision éventuelle pour le patient :

Déviation	***Décision éventuelle pour le patient***

DECES

Si décès du patient,

- Date de décès : I__I__I / I__I__I / I__I__I__I__I
- Cause du décès : ___________________________

Je soussigné, Pr/Dr …………………………,
Investigateur, certifie exactes les données recueillies dans ce cahier d'observation et les corrections faites conformément aux données du dossier médical.

Date : I__I__I / I__I__I / I__I__I__I__I

Signature :
